# Antecedents of Employee Green Behavior in the Hospitality Industry

**DOI:** 10.3389/fpsyg.2022.836109

**Published:** 2022-06-29

**Authors:** Noor Ullah Khan, Jie Cheng, Muhammad Yasir, Roselina Ahmad Saufi, Noorshella Che Nawi, Hanieh Alipour Bazkiaei

**Affiliations:** ^1^Malaysian Graduate School of Entrepreneurship and Business (MGSEB), Universiti Malaysia Kelantan (UMK), Pengkalan Chepa, Malaysia; ^2^Department of HRM, NUST Business School, National University of Sciences and Technology, Islamabad, Pakistan; ^3^School of International Education Anhui Xinhua University Hefei, Anhui, China; ^4^Department of Management Sciences and Commerce, Bacha Khan University, Charsadda, Pakistan; ^5^Global Entrepreneurship Research & Innovation Centre (GERIC), Universiti Malaysia Kelantan, Kota Bharu, Malaysia; ^6^Faculty of Entrepreneurship and Business, Universiti Malaysia Kelantan (UMK), Pengkalan Chepa, Malaysia

**Keywords:** environmental-specific ethical leadership, psychological green climate, employees’ green behavior, hospitality industry, structural equation modeling

## Abstract

Organizations are increasingly adopting green human resource management policies to encourage environmentally friendly behaviors. Research shows that adopting green policies and procedures is beneficial for the hospitality industry. However, limited empirical evidence exists on the association between environmental-specific ethical leadership, psychological green climate, and employees’ green behavior. Therefore, this study intends to examine psychological green climate (PGC) as a mediator between the relationship of environmental-specific ethical leadership (ESEL) and employees’ green behavior (EGB), specifically in the hotel industry of Pakistan. Data from 224 non-managerial position employees in the understudy sector was collected using a convenient sampling technique. Structural equation modeling (SEM) was utilized to examine the direct and indirect effects among the variables using the Smart PLS 3.3.3 version. This study showed that ESEL is positively related to PGC and EGB. Moreover, PGC is positively associated with EGB, and PGC mediated in the relationship between ESEL and EGB. Thus, current research highlights the significance of environmental-specific ethical leadership behavior, which assists in establishing a green psychological climate, thereby fostering employees’ green behavior in the hotel industry of Pakistan.

## Introduction

Researchers are recently interested in the role of organizational environmental sustainability, thereby encouraging employee’s green behavior at the workplace (Norton et al., 2014; Stalcup et al., 2014; Zhou et al., 2018; Gurmani et al., 2021; Khan et al., 2021a) However, scholars view that the hotel industry has demonstrated less emphasis on environmental issues for instance employees’ green behavior (Okumus et al., 2019; Arshad et al., 2021; Khan et al., 2021b). In addition, studies show that adopting green procedures and practices benefits the hospitality sector (Gil et al., 2001; Blanco et al., 2009; Chou, 2014) through innovations, competitive advantages (Goodman, 2000), customer satisfaction, and loyalty (Kassinis and Soteriou, 2003). Specifically, the hotels’ success in adopting green procedures not only depends upon organizational policies regarding green practices but also on its employees’ support of environmental protection (Chou, 2014). Thus, employees play an essential role who is assisting in the implementation of green organizational policies and procedures; therefore, an organization must foster and eventually change employees’ attitudes and behavior so that such behavior is in line with the green goals of the organization (Ramus and Steger, 2000; Daily et al., 2009; Ones and Dilchert, 2012; Dumont et al., 2017). Research shows that the employees’ behavior makes a significant contribution to cost-saving and waste reduction (Tam and Tam, 2008), competitive advantage (Del Brío et al., 2007), and organizational environmental performance (Boiral et al., 2015b). Despite these investigations, studies that show the antecedents of employees’ green behavior are still in their early stages (Dumont et al., 2017; Robertson and Carleton, 2018; Zientara and Zamojska, 2018), specifically in the developing countries like Pakistan (Saleem et al., 2020), and in the hospitality sector of Pakistan (Ahmed et al., 2020; Arshad et al., 2021).

Green behavior at work is defined as “a broad set of environmentally-responsible activities such as learning more about the environment, developing and applying ideas for reducing the company’s environmental impact, developing green processes and products, recycling and reusing, and questioning practices that hurt the environment” (Graves et al., 2013). Previous literature has emphasized the need to investigate the individual and contextual predictors of employees’ green behavior and the mechanisms through which several contextual and personal factors can affect employees’ green behavior (Norton et al., 2015, 2017). Therefore, scholars are increasingly interested in the leadership role as a critical antecedent of employees’ green behavior (Robertson and Barling, 2013; Afsar et al., 2016; Kura, 2016; Robertson and Carleton, 2018; Gurmani et al., 2021). Thus, literature shows a growing call for investigating leadership behavior that promotes subordinate’s green workplace behavior, thereby minimizing organizations’ adverse effects on the environment (Boiral et al., 2015b; Khan et al., 2019; Liu and Zhao, 2019; Saeed et al., 2019; Suganthi, 2019).

The current study analyzes the association between environmental-specific ethical leadership and employees’ green behavior. Brown et al. (2005) have defined ethical leadership as “the demonstration of normatively appropriate conduct through personal actions and interpersonal relationships, and the promotion of such conduct to followers through two-way communication, reinforcement, and decision making” (p: 120). Thus, an environmental-specific ethical leadership style fundamental focus on ethics (Brown et al., 2005) will have the ability to influence environmental sustainability and employees’ pro-environmental behavior (Khan et al., 2019; Saleem et al., 2020).

Previous literature shows that the individuals’ perception of green climate positively affects the green behaviors of the employees (Dumont et al., 2017; Norton et al., 2017; Khan et al., 2019). Previous literature also shows that a green climate is one of the psychological mechanisms through which various leadership styles are associated with pro-environmental behavior and performance (Kura, 2016; Zhou et al., 2018; Khan et al., 2019; Tuan, 2021). However, the knowledge and understanding regarding the psychological mechanisms through which organizations can inculcate employees’ green behavior are still in their infancy (Saleem et al., 2020). Therefore, this study attempts to analyze the psychological green climate as a mediator between environmental-specific ethical leadership and employees’ green behavior. Dumont et al. (2017) view psychological green climate as “the perception an individual has of the organization’s pro-environmental policies, processes, and practices that reflect the organization’s green values” (p: 4).

As the success of environmental actions in organizations mainly depends upon the employees’ behaviors (Daily et al., 2009; Paillé et al., 2013; Gurmani et al., 2021), so, employees should be encouraged to practice green workplace behavior, thereby addressing the growing environmental concerns (Ones and Dilchert, 2012; Paillé and Boiral, 2013; Kura, 2016). In addition, it is necessary to understand how employees will engage in green workplace behavior. Therefore, the current research extends the limited literature regarding the effect of environmental-specific ethical leadership on employees’ green behavior. Secondly, the recent research highlights psychological green climate as one of the underlying psychological mechanisms for the relationship between environmental-specific ethical leadership and employees’ green behavior. Thirdly, the current study extends the limited literature on employees’ green behavior by offering two key antecedents.

### Industry Background

Pakistan is likewise becoming a popular investment destination for new hotel development. In terms of occupancy and average rate, hotels in Pakistan are experiencing significant expansion. In the following years, this trend is projected to continue. Tourism is an integral part of Pakistan’s economy and a major source of foreign exchange earnings. Pakistan’s government has relaxed its visa policy, which has helped the country attract many foreign visitors. Over the last 6 years, 60,070 foreign visitors have visited the country’s tourist attractions, particularly the captivating sites found in the country’s northern regions. According to the present government’s plans and steps to boost domestic and international tourism, the country forecasts a nearly 30% increase in visitors between now and 2030. Pakistan received approximately 948 million dollars in global tourism receipts in 2019. Although tourism receipts in Pakistan have fluctuated significantly in recent years, they have climbed from 2015 to 2019. Hilton struck a deal with Dhabi Hospitality in March 2021 to build an upscale Doubletree by Hilton hotel in Islamabad, Pakistan, that is expected to open in 2025. The Radisson Hotel Group and Radisson Blu Serviced Apartments in Islamabad, Pakistan, struck a deal in August 2020 to open the country’s first internationally branded serviced apartments (Intelligence, 2022).

In 2020, the Pakistan tourist and hotel market was estimated to be worth roughly USD 20 billion, with a CAGR of 3% predicted by 2026. In 2019, tourism contributed about 7% of Pakistan’s gross domestic product (GDP). However, the market has suffered significant losses because of the pandemic. People cannot visit Pakistan due to safety precautions and different lockdowns implemented by the government during the current COVID-19 pandemic, putting Pakistan’s tourist and hospitality sectors in danger. The Pakistani government and people believed that the development would spur greater investment in tourism-related enterprises and create higher-paying jobs. The administration had also declared plans to promote and enhance tourism and remove visa rules for foreign visitors. The commencement of COVID-19 and restrictions on international travel have hampered the government’s plan; as a result, the country will not be able to implement its tourist strategy in 2020 (Intelligence, 2022). On the other hand, the market is likely to gain traction in the long to medium term. Over the last few years, Pakistan has become one of the most popular tourist destinations globally.

Furthermore, the South Asian country has topped several worldwide travel lists, making it a popular tourist destination. The government of Pakistan’s tourism promotion has also aided in raising the country’s profile in the international market. Increased improvements in roads, airports, and other facilities have helped ease travel. In Pakistan, the tourism and hotel industry are divided into two categories: inbound and outbound tourism, as well as hotel types [economy and budget hotels, mid-scale hotels, upper-scale hotels, premium and luxury hotels, and other types of hotels (shared living spaces, rented apartments, service apartments, and so on)]. For all of the following segments, the research provides market size and projections in USD million for Pakistan’s tourist and hotel sector (Intelligence, 2022).

**Figure 1:**
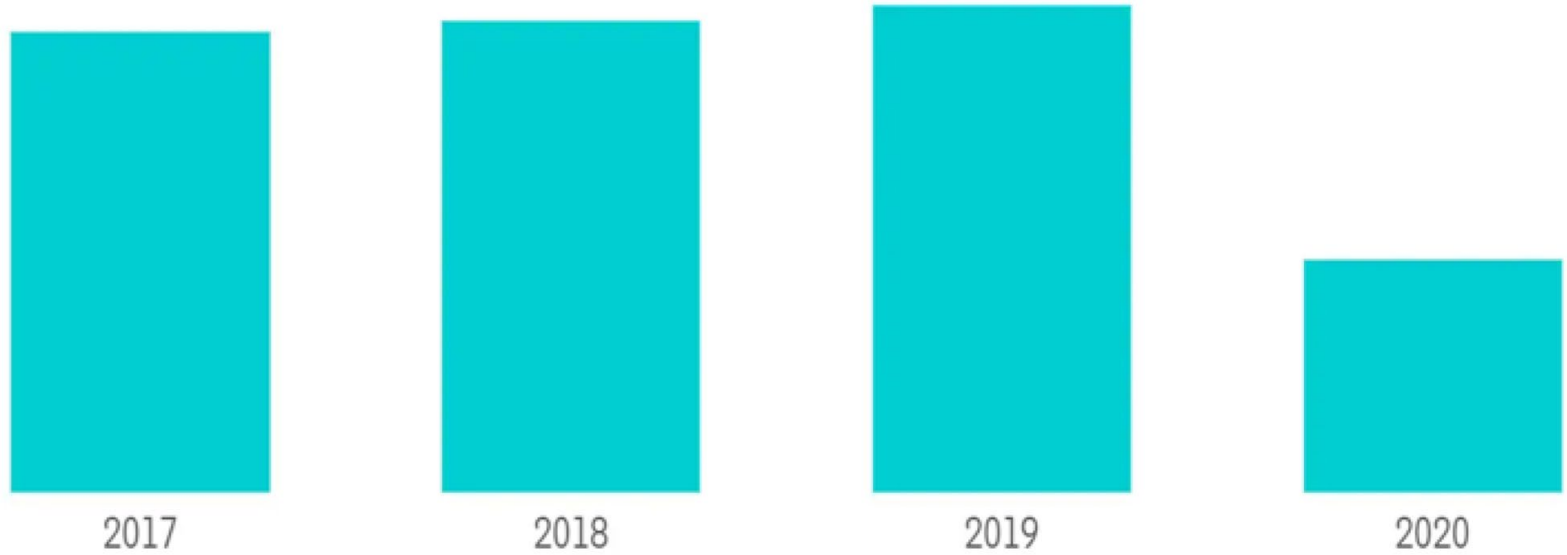
Pakistan’s Hotel Industry Revenue in Millions of Dollars (2017–2020). Source: (Intelligence, 2022).

**Figure 2:**
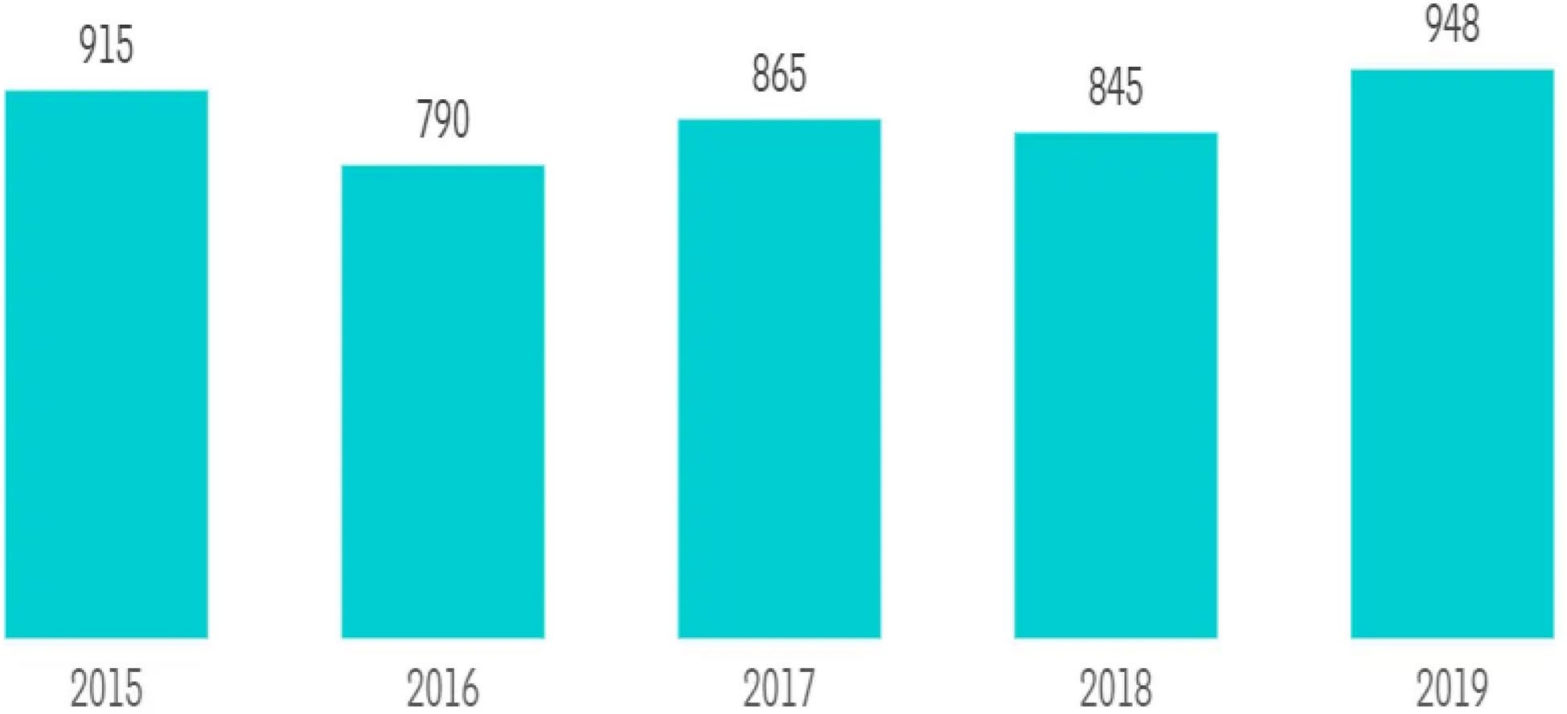
Pakistan’s international tourism receipts, in millions of dollars (2015–2019). Source: (Intelligence, 2022).

The hospitality sector in Pakistan, a developing country, was the focus of this research. We chose the hotel segment in the country to represent the hospitality sector because it is a mix of major five-star hotels, such as Serena, Avari, Marriot, and Pearl Continental, which are some of its top competitors. In recent years, Pakistan has become an important investment destination for the hospitality and tourism sector, particularly for new hotel development. Indeed, the tourism and hotel sector’s net worth were close to 20 billion dollars in 2020. Given the significant expansion in this area in recent years, 262 expects a CAGR of more than 3% in 2026. From an economic standpoint, these patterns are favorable for the country’s GDP (see, for example, Figures 1 and 2). However, the increased competition in this sector is reflected in the growth trends. Every hotel in the country is concerned about how to survive and remain competitive in the face of competition. In this regard, increasing staff creativity is critical in this industry—large cities such as Lahore, Karachi, Faisalabad, Islamabad, and Rawalpindi are important destinations for hotel investment.

In the hospitality industry, just a little contemporary research has been undertaken. A recent study looked at the impact of ethical leadership on people’s green in-role and extra-role behaviors, with green HRM practices as a mediating factor and individual green values as a moderator. The findings revealed that ethical leadership considerably impacts green HRM practices and in-role and extra-role green behaviors. Furthermore, the association between ethical leadership and both types of green behaviors was mediated by green HRM practices. Furthermore, individual green values were found to improve the link between green HRM practices and both types of green behaviors (Islam et al., 2021b). Another study looked at the impact of a supervisor’s ethical leadership style on subordinates’ green or pro-environmental work behavior in the presence of green human resource management (GHRM) as a mediator and environmental knowledge as a moderator, as well as the influence of a supervisor’s ethical leadership style on subordinates’ green or pro-environmental work behavior. According to structural equation modeling, the influence of ethical leadership on green work behavior was partially mediated by GHRM.

Similarly, a recent study investigated the impact of green human resource management in mediating the relationship between ethical leadership and employees’ environmental citizenship behavior and the moderating effect of individual green ideals. The findings revealed that ethical leadership directly impacts employees’ environmental citizenship behavior and an indirect impact through green human resource management. Furthermore, the results demonstrated that, based on social learning and supply value-fit theory, employees’ environmental knowledge might multiply the indirect impact of ethical leadership on green behavior *via* GHRM (Islam et al., 2021a).

The current project attempts to close the following knowledge gaps. First, to our knowledge, this is the first viewpoint that tries to promote ESEL as a crucial antecedent and the mediating effects of PGC in predicting employee green behavior, which has not been examined previously. Second, from an individual standpoint, this effort tends to enhance the topic of environmental-specific ethical leadership in the hotel industry context. This industry plays a significant role in corporate social responsibility at the employee level, servant leadership, and innovative work habits (Ahmad et al., 2021a). In this vein, most of the previous study in the domain of innovation was conducted at the organizational level, for example (Ahmad et al., 2021b; Islam et al., 2021a). Despite the importance of individual green behavior and ESEL through PGC, the hotel industry has been understudied. Finally, numerous ESEL studies have been undertaken in developed countries. Given that ESEL is influenced by culture, climate, and context-specific scenarios (Slåtten et al., 2020).

The remainder of the current manuscript is separated into various sections. Next, research objectives followed by the research model, theoretical underpinnings, and relevant literature will be explored. Next, the authors present the methodology, including information on the population, sample, and data gathering technique. The results and analyses for validating the hypotheses are discussed in the fourth part. Implications, limitations, and conclusions are also included in this section. Finally, in the discussion section, the authors examine their findings from past research.

### Research Objectives

The objectives of this study are to (i) determine the relationship between environmental-specific ethical leadership and employees’ green behavior, (ii) investigate the effect of environmental-specific ethical leadership on psychological green climate, and (iii) examine the effect of psychological green climate on employees’ green behavior, and (iv) to investigate the mediating role of psychological green climate between the relationship of environmental-specific ethical leadership and employees’ green behavior in the context of Pakistan’s hotel industry.

### Research Model

**Figure 3:**
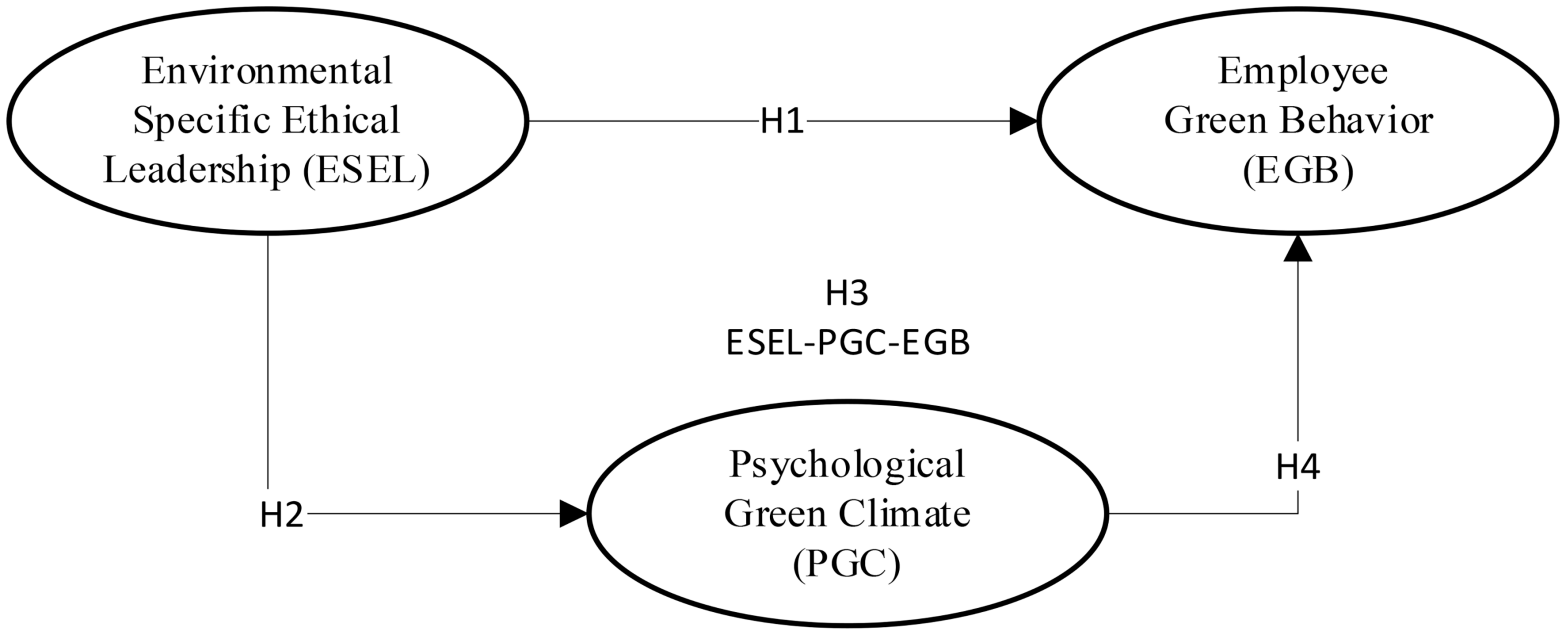
Research model.

This research has three primary constructs, instance, ESEL (exogenous variable), PGC (mediator), and (3) EGB (endogenous variable; see Figure 3). This study model is based on the social exchange theory (Blau, 1964; Cropanzano et al., 2017) and the ability-motivation-theory (Appelbaum, 2000) and social learning theory as a theoretical foundation discussed in detail in literature section.

## Literature Review on Ethical Leadership

The contemporary work environment is shaped by increasing global connectivity and integration, which requires an organic and holistic leadership style (Crews, 2010). Therefore, ethical leadership is necessary to achieve organizational success (Mccann and Sweet, 2014; Ko et al., 2018). As ethical leadership positively influences several employees’ job outcomes, for instance, commitment, satisfaction, and performance (Kim and Brymer, 2011; Ahn et al., 2016; Özden et al., 2019), and negatively influence work outcomes, for instance, job stress, burnout, and workplace deviance (Schwepker and Ingram, 2016; Lin and Liu, 2017; Yasir and Rasli, 2018). Brown et al. (2005) categorized ethical leadership into two main components: (1) moral manager (role modeling ethical behavior, rewarding/punishing, emphasizing ethical standards), and (2) moral person (fairness, integrity, trustworthiness, concern for others). Ethical leaders are those individuals who have a genuine concern regarding how their decisions may affect the well-being of their followers (Brown and Treviño, 2006; Northouse, 2007). Therefore these leaders tend to care more about their subordinates, society, and organization than their self-interests (Bachmann, 2017; Ko et al., 2018).

### Employee Green Behavior

Recently researchers have been increasingly interested in studying green workplace behavior (Norton et al., 2015; Kura, 2016; Dumont et al., 2017; Suganthi, 2019). The attention provided to employees’ green behavior emerged from the need for employees to protect the environment and utilize resources wisely (Kura, 2016). Ones and Dilchert (2012) view employees’ green behavior as “scalable actions and behaviors that employees engage in that are linked with and contribute to or detract from environmental sustainability” (p: 87). Green behavior of the employees includes activities, for instance, editing documents electronically instead of printouts, reporting leaks in the toilet, using teleconference facilities instead of traveling to meetings, turning off unwanted lights, and printing drafts on scrap papers (Norton et al., 2015, 2017). Thus, the green behavior of the employees focuses on avoiding waste, conserving water, saving energy, and recycling. Therefore, scholars view that the green behavior of an employee is an individual’s commitment which can be stimulated through encouragement rather than through demands (Pichel, 2008). Hence, contextual, cultural, and individual factors significantly shape employees’ green behavior (Pichel, 2008; Chou, 2014).

### Psychological Green Climate

An individual’s psychologically meaningful representations of proximal organizational processes, events, and structures are a psychological climate (Rousseau, 1988). It is also defined as “an experiential-based, multidimensional, and enduring perceptual phenomenon which a given organizational unit members widely share. Its primary function is to cue and shape individual behavior toward the modes of behavior dictated by organizational demands” (Koys and DeCotiis, 1991). Several kinds of psychological climates exist in the previous literature (Yasir et al., 2017). One of them is a green climate which is defined as a shared perception of the organizational policies, procedures, and practices that are established and promoted, thereby pursuing a sustainable environment (Chou, 2014; Norton et al., 2017; Zhou et al., 2018; Tuan, 2021). Moreover, Chou (2014) identified that the green climate dimensions include management orientation and environmental policy, for instance, training and policy statements, to specific ecological tasks, for example, water resource management and recycling. Thus, the formation of the climate is influenced by the degree of organizational emphasis on particular procedures and practices (Kuenzi and Schminke, 2009).

#### Theoretical Foundations AMO Theory

According to Appelbaum (2000), the AMO theory describes the components of high-performance work systems, which stands for ability (selective hiring, training, education, and development of talented staff), motivation (incentive system, performance-based payments), and opportunity (selective hiring, training, education, and development of talented team; involving employees in EM initiatives). Performance, according to the AMO theory, is the outcome of the interaction of employees’ ability to perform (ability), willingness to perform (motivation), and opportunity to perform (participation; opportunity). Previous research applied AMO theory to green HRM practices and environmental initiatives, such as identifying and developing employees’ green competencies; developing a system of green performance appraisal and green rewards that generate green motivation; and providing employees with ways to operate with flexibility, autonomy, and participation in decision making, all to increase employee green behaviors in the workplace (Renwick and Redman, 2013). An organization employs the AMO framework to inspire employees through green rewards and provide employees with green opportunities to improve their performance, resulting in increased productivity, quality, organizational performance, waste reduction, and profit (Renwick and Redman, 2013). HRM practices impact employee attitudes and behavior at both the individual and organizational levels. Furthermore, more than half of all publications published after 2000 have used AMO theory to green HRM practices and green behaviors, including pro-environmental behaviors, OCBE, and other green initiatives (Paauwe and Boselie, 2005; Paauwe, 2009).

#### Social Exchange Theory

Nearly half a century ago, Homans (1958) proposed a paradigm of social behavior centered on exchange. The exchange of activity, material or intangible, and rewards between at least two parties,” Homans defined “social exchange. Social exchange refers to voluntary behaviors of individuals motivated by the returns they are expected to bring and normally do bring from others,” writes Blau (1964). A more formal psychological underpinning for exchange was constructed based on these basic reinforcement principles. Relationships can be ended due to a lack of reinforcement or an excessive disparity in the appropriate incentives and costs. Similarly, positive-results-producing conduct is more likely to be repeated and rewarded (Blau, 1964; Emerson, 1976). The social exchange theory (SET) is one of the most significant conceptual models for analyzing workplace behavior (Cook et al., 2013). When anything regarded as the worth is traded between an organization and its personnel, it is referred to as the social exchange. As previously stated, implementing SET helps to create an environment in which employees are more inclined to reciprocate by exhibiting behaviors that the company values. Employees must be aware that their organization is serious about environmental sustainability to reciprocate appropriately, i.e., by engaging in green behavior. According to researchers, employees may engage in behaviors, deeds, or gestures that reflect their acceptance of the organization’s desired purpose on a voluntary rather than mandated basis. Similarly, SET reveals that employees at companies are more likely to engage in green behavior if they feel encouraged (Boiral et al., 2015a) based on reciprocity norms. Employee green behavior might be seen as repayment in exchange for organizational support in this setting.

Furthermore, this literature on green practices in firms is based on SET, whose fundamental principle is employee-organization reciprocity. According to SET, employees will be more willing to engage in green behavior to reciprocate the benefits if they are aware that environmental protection is an essential goal and if they feel encouraged by the company. As a result, employees can engage in green behavior at work if they believe their company values environmental sustainability alongside economic objectives that each party receives from the other in the exchange process (Paillé et al., 2016; Raineri et al., 2016; Khan et al., 2021a,b).

Nonetheless, the social transaction generates a bond between employees and their employers that extends beyond economic goals (Blau, 2017) claimed that the process of social exchange could not be reduced to a single issue since the idea is so broad that it encompasses a wide range of issues. This idea was also used in the early work of Choi et al. (2015) and Qi et al. (2019) to explain how the social interchange between an inclusive leader and a follower encourages him to engage in extra-role behaviors, such as creative behavior. An inclusive leader develops an environment of justice, trust, respect, and collaboration with followers through encouraging openness in the workplace. Furthermore, an inclusive leader helps employees by resolving legal issues. At the same time, such leaders assist their employees in situations not covered by an employee’s legal contract. Employees are encouraged to provide more support to their inclusive leader in the social exchange process when they witness their supportive and caring behavior. As a result, individuals can engage in various extra-role activities, one of which is their creative potential.

#### Social Learning Theory

We also use the term “social learning theory” in this context. We also mention social learning theory, which explains why employees engage in creative jobs due to the social learning process. This hypothesis, developed by (Bandura and Walters, 1977), contends that individuals’ social behaviors are shaped by seeing the actions of others. Individuals replicate the activities of others in a specific situation when they watch them. Leaders play a critical role in influencing employee behavior in the workplace. In the contemporary environment, an inclusive leader supports an open culture in an organization. Not only does he urge his followers to come up with fresh ideas for creatively performing tasks, but they also emulate his ingenuity. When employees observe this type of leadership behavior, social learning assists them in learning it so that they may put it into practice on their own. Scholars have mostly looked at leadership studies to explain how employees learn specific behaviors from their boss through a social exchange process (Robertson and Carleton, 2018; Bai et al., 2019).

### Environmental-Specific Ethical Leadership and Employees’ Green Behavior

The leadership of an organization influences various work outcomes, for instance, employee attitudes (Barling et al., 2011), environmental performance (Ramus and Steger, 2000), and safety performance (Barling et al., 2002). Prior literature shows that situational and individual factors can affect individuals’ probability of engaging in green workplace behavior (Kura, 2016). For example, previous literature identified that transformational leadership behavior and spiritual leadership behavior play a significant role in maximizing green workplace behavior (Graves et al., 2013; Robertson and Barling, 2013; Afsar et al., 2016; Kura, 2016). Research studies show that when employees work under the leadership of those individuals who display servant leadership behavior that focuses on protecting the environment, they tend to exhibit green behaviors more often (Afsar et al., 2018; Tuan, 2021). Recently, Liu and Zhao (2019) study identified that when subordinates perceive that in their organization, ethical leadership focuses upon sustainable development, cares about others, and possesses the values of green production and green research and development, thus affecting subordinates’ green innovation behavior. Therefore, the current study proposes the following hypothesis.

*Hypothesis 1*: Ethical leadership is positively related to employees’ green behavior.

### Environmental-Specific Ethical Leadership and Psychological Green Climate

Stringer (2002) highlighted that “most studies have shown that the single most important determinant of an organization’s climate is the day-to-day behavior of the leaders of the organization” (p: 12). More specifically, climate formation is influenced by the employee’s immediate supervisor. Because employees view and follow the actions of their immediate supervisors (Cantor et al., 2012). Therefore, supervisors should explain to their followers the importance of a specific organizational initiative and act as an interpretive filter of relevant organizational policies and procedures (Kuenzi and Schminke, 2009), and provide them with a source of inspiration, for instance, ethical leadership behavior (Zientara and Zamojska, 2018). As sustainability is an ethical issue (Barnett et al., 2005), an ethical leader is highly likely to assist in developing and promoting environmental standards to protect the natural environment (Khan et al., 2019). Moreover, an ethical leader encourages their subordinates to raise their concerns regarding the ethical standards (Yasir and Rasli, 2018), they are also expected to promote discussions between followers related to the environmental standards (Kuenzi and Schminke, 2009), thereby shaping subordinates shared perception that the organization’s environmental procedures and policies are established to improve environmental sustainability (Khan et al., 2019; Saleem et al., 2020). Hence, current research proposes that ethical leadership will positively and significantly associate with the psychological green climate.

*Hypothesis 2*: Ethical leadership is positively related to a psychological green climate.

### Psychological Green Climate and Employees’ Green Behavior

Previous literature shows that contextual factors can affect employees’ green behavior (Norton et al., 2012; Littleford et al., 2014; Dumont et al., 2017). More specifically, prior studies show that organizational climate is a significant contextual factor influencing employees’ behavior and attitude (Kuenzi and Schminke, 2009; Yasir and Rasli, 2018). As Norton et al. (2012) identified that green climate captures individuals’ perceptions regarding the behavioral norms and organizational attributes within an organization that pertain to environmental sustainability. Therefore, these scholars argue that a green climate is necessary to establish, thereby facilitating an employee’s green behavior. In line with the previous literature on psychological climate (James et al., 2008), the psychological green climate has a positive association with the green behavior of the employees (Norton et al., 2014; Dumont et al., 2017). Previous literature also shows that a green climate predicts next-day employees’ green behavior (Norton et al., 2017) because individuals are primarily motivated to exhibit behaviors in line with their perceptions of their organization’s procedures and practices (Schneider, 1975). Thus, they might believe that exhibiting green behavior is appropriate in their organization (Norton et al., 2014). Recently, Saleem et al. (2020) identified a positive association between green climate and employees’ green behavior in universities and hospitals in Pakistan. Hence, it is proposed that a psychological green climate will have a positive and significant association with employees’ green behavior.

*Hypothesis 3*: The psychological green climate is positively related to employees’ green behavior.

### Mediating Role of Psychological Green Climate

Past literature shows that ethical leaders can promote and develop environmental standards to protect the natural environment (Khan et al., 2019). By adopting green human resource management practices, a message is conveyed by the organization’s top management to the employees regarding its concern for a sustainable work environment beyond pure economic gains. Further, it seeks to engage employees in green-related activities and decisions (Renwick and Redman, 2013). Moreover, if top management of an organization clarifies green responsibilities in the workplace, for instance, having appropriate rewards for green behavior, proper job design, and appraisal, thereby enhancing employees’ awareness of a sustainable environment leading toward their involvement in green activities (Dumont et al., 2017).

Previous research also indicates the mediating role of green climate between (a) perceived organizational sustainability policy and employees’ green behavior (Norton et al., 2014), (b) environmental-specific servant leadership and green performance (Tuan, 2021), and (c) ethical leadership and organizational environmental citizenship behavior (Khan et al., 2019). Therefore, it is proposed that a psychological green climate mediates the association of ethical leadership and employees’ green behavior in Pakistan’s hotel industry.

*Hypothesis 4*: The psychological green climate mediates the relationship between ethical leadership and employees’ green behavior.

## Methodology

This study aimed to see how ESEL and PGB affected EGB in the Pakistani hotel business. This study used a logical approach and a quantitative technique based on the positivist worldview. The primary data was acquired using conventional survey instruments from previous studies and was cross-sectional. The responders were able to comprehend all of the questionnaire items. Part, one consisted of demographic questions such as age, gender, marital status, educational background, the highest degree of schooling, work experience, duration of service, and department. The second section included questions about three constructs: ESEL, PGC, and EGB. The quantitative technique stresses numerical data, demands random sampling, and requires the research topics to be predetermined. It entails structured data collection instruments such as questionnaires.

It is concerned with the outcomes of the study being generalized. As a result, a survey design was selected, and individually administered questionnaires were used (Creswell and Creswell, 2017). According to the literature on sustainability, upmarket hotels are on the cutting edge of ecologically friendly measures (Merli et al., 2019). Pakistan’s hotel business contributes to the country’s Gross Domestic Product (GDP) while also providing a considerable number of job opportunities (WTTC, 2020). One of the researchers contacted the managers of the five-star hotels for data collection, and the managers agreed to enable their personnel to complete the questionnaires. As a result, data from five-star hotels was obtained using the probability sampling method. The survey was given by hand, and respondents were informed that their personal information would be kept private and used solely for research purposes.

### Target Population and Sample Size

According to the literature on sustainability, upmarket hotels are on the cutting edge of ecologically friendly measures (Zientara and Zamojska, 2018; Merli et al., 2019). As a result, five-star hotels were chosen as the example location. The combined total headcount of three five-star hotels was roughly 1,592 at the time of data collection. A 310 respondents were computed from a population frame of 1,592 using the formula *n* = [*z*^2^ * *p* * (1 − *p*)/*e*^2^]/[1 + (*z*^2^ * *p* * (1 − *p*)/(*e*^2^ * *N*)] The required sample size was determined to be 310 at a 95 percent confidence level and a 5 percent margin of error (Daniel, 1999) and sampling Table by Krejcie and Morgan (1970). The sample size is 310 (with finite population adjustment).


$$n=\left[z^{2*}\;p^{*}\;\left(1-p\right)/e^{2}\right]/[1+(z^{2*}\;p^{*}\;\left(1-p\right)/\left(e^{2*}\;N\right)]\text{,}$$

where *z* = 1.96 indicates a 95% confidence level, *p* = proportion (represented as a decimal), *N* = population size, and *e* = margin of error.

*z* = 1.96, *p* = 0.5, *N* = 1,592, *e* = 0.05


$$\begin{array}{l} n=\left[1.962^{*}\;0.5^{*}\;\left(1–0.5\right)/0.052\right]/\\ \;[1+\left(1.962^{*}\;0.5^{*}\;\left(1–0.5\right)/\left(0.052^{*}\;1592\right)\right] \end{array}$$

*n* = 384.16/1.2413 = 309.48

*n* ≈ 310

Although, according to the methodology, the required sample size was 310. Maintaining a low response rate, on the other hand, is a delicate topic for organizational researchers. Some academics feel that a response rate of more than 50% is sufficient for research to confirm results (Hair et al., 2010a; Saunders et al., 2012; Hiebl and Richter, 2018). As a result, researchers must recognize the significance of a fair response rate for their studies. Based on this perspective, the current study aimed for a significantly bigger sample size to prevent low response rate difficulties and obtain the necessary data for SEM analysis.

### Data Collection and Response Rate

Self-administered questionnaires were used. The researcher first sent 350 self-administered questionnaires to five-star hotel personnel. A total of 238 questionnaires were returned, suggesting a 68 percent response rate. For generalizing the results, a response rate of more than 50% is required (Sekaran and Bougie, 2016). However, normality tests revealed that 14 of the patients had concerns with normality. As a result, after excluding 14 cases, the final data of 224 respondents was used, meeting the needed minimum sample size for SEM of 200 responses (Hair et al., 2010a). Simple random sampling was used in this investigation. The researcher selects the individuals at random using this technique, eliminating human biases. This method ensures that each participant has an equal probability of being included in the survey (Creswell and Creswell, 2017).

### Measurement Instruments

Employees were asked to provide their opinions regarding each construct’s understudy constructs and items. For instance, ethical leadership, employees’ green behavior, and psychological green climate was anchored on a 5-point Likert scale ranging from “1 = strongly disagree to 5 = strongly agree.” The survey had four parts. Part, one had questions related to the demographic description, such as age, gender, marital status, educational background, highest education level, work experience, length of service, and department. The second part had questions related to ethical leadership behavior and was measured using a ten-item scale from Brown et al. (2005). The third part had questions related to employees’ green behavior and was measured using 13 items scale from Graves et al. (2013). Lastly, part fourth had questions related to psychological green climate and was measured using eight items from Norton et al. (2014).

## Data Analysis and Results

Descriptive statistics are composed of several demographic variables: age, gender, marital status, educational background, highest education level, work experience, length of service, and department. Table 1 highlights the frequency and percentages of each demographic variable.

**Table 1:** Demographic characteristics.

Demographic characteristics	Frequency	Percentage
Age		
18–24	45	20.1
25–30	116	51.8
31–35	34	15.2
36–40	16	7.1
41–45	08	3.6
46–50	05	2.2
Total	224	100.0
Gender		
Male	171	76.3
Female	53	23.7
Total	224	100.0
Marital status		
Single	99	44.2
Married	125	55.8
Total	224	100.0
Educational background		
Arts	107	47.8
Sciences	08	3.6
Management	53	23.7
Engineering/IT	07	3.1
Hospitality	47	21.0
Other	02	0.9
Total	224	100.0
Highest education degree		
Metric	03	1.3
Intermediate	28	12.5
Bachelors (14 years)	77	34.4
Bachelors (16 years)	21	9.4
Masters (16 years)	18	8.1
Masters (18 years)	31	13.8
Diploma	46	20.5
Total	224	100.0
Work experience		
1–3	42	18.9
4–6	75	33.6
7–9	39	17.5
10–12	26	11.6
13 and beyond	41	18.4
Total	224	100.0
Length of service		
Less than 1	04	1.8
1–3	143	63.8
4–6	58	25.9
7–9	14	6.3
10–12	04	1.8
13 and beyond	01	0.4
Total	224	100.0
Department		
Housekeeping	64	28.6
Food and beverages	49	21.9
Human resource	06	2.7
Account/Finance	11	4.9
Sales/Marketing	12	5.4
Reception	05	2.2
Rooms	09	4.0
Kitchen	43	19.2
Security	08	3.6
IT/Engineering	07	3.1
Quality and compliance	01	0.4
Laundry	02	0.9
Material management	01	0.4
Other	06	2.7
Total	224	100.0

Initially, the researcher distributed 350 self-administrated questionnaires among employees of the five-star hotels. In response, 238 questionnaires were returned, indicating a response rate of 68%. A response rate greater than 50% is appropriate for generalizing the results (Sekaran and Bougie, 2016). However, normality tests showed that 14 cases had normality issues. Therefore, after removing 14 cases, the final data of 224 respondents was used, which fulfills the required minimum sample size for SEM, which is 200 responses (Hair et al., 2010a).

### Measurement Model

In Smart PLS, the estimation of the research model is based on a two-stage firstly valuation of the measurement model by investigating its reliability and validity, and secondly, a structural model that examines the variance explanation of the endogenous construct and predictive relevance. Many researchers have recommended applying a measurement model to establish the reliability and validity of the primary data. Three-step procedures, namely, individual item reliabilities, convergent validity, and discriminant validity, are required to assess the measurement model. Mostly the recommended “threshold limits of the factor loadings should be higher than 0.70” (Hair et al., 2006, 2011, 2012). Table 2 shows the item loadings for understudy constructs which assess the convergent validity.

**Table 2:** Items loading.

Item	ESEL	EGB	PGC
My manager listens to what employees have to say.	0.737		
My manager conducts his/her personal life in an ethical manner.	0.767		
My manager defines success not just by results but also the way that they are obtained.	0.770		
My manager disciplines employees who violate ethical standards.	0.797		
My manager makes fair and balanced decisions.	0.768		
My manager can be trusted.	0.719		
My manager discusses business ethics or values with employees.	0.755		
My manager sets an example of how to do things the right way in terms of ethics.	0.726		
My manager has the best interests of employees in mind.	0.822		
My manager, when making decisions, asks, “what is the right thing to do?”	0.817		
I try to learn more about the environment.		0.728	
I find ways of working that are better for the environment.		0.763	
I offer ideas for reducing our impact on the environment.		0.76	
I share my knowledge about the environment with others.		0.767	
I apply new ideas for reducing our impact on the environment.		0.767	
I help create green processes and products.		0.722	
I perform environmental tasks that are not required by my company.		0.724	
I question practices that are likely to hurt the environment.		0.76	
I recycle and reuse materials.		0.783	
I try to reduce my energy use.		0.800	
I join in environmental activities that are not required by my job.		0.770	
I encourage others to think about the environment.		0.701	
I help others solve environmental problems.		0.674	
Our hotel is worried about its environmental impact.			0.815
Our hotel is interested in supporting environmental causes.			0.728
Our hotel believes it is important to protect the environment.			0.704
Our hotel is concerned with becoming more environmentally friendly.			0.759
In our hotel, employees pay attention to environmental issues.			0.76
In our hotel, employees are concerned about acting in environmentally friendly ways.			0.838
In our hotel, employees try to minimize harm to the environment.			0.846

**Figure 4:**
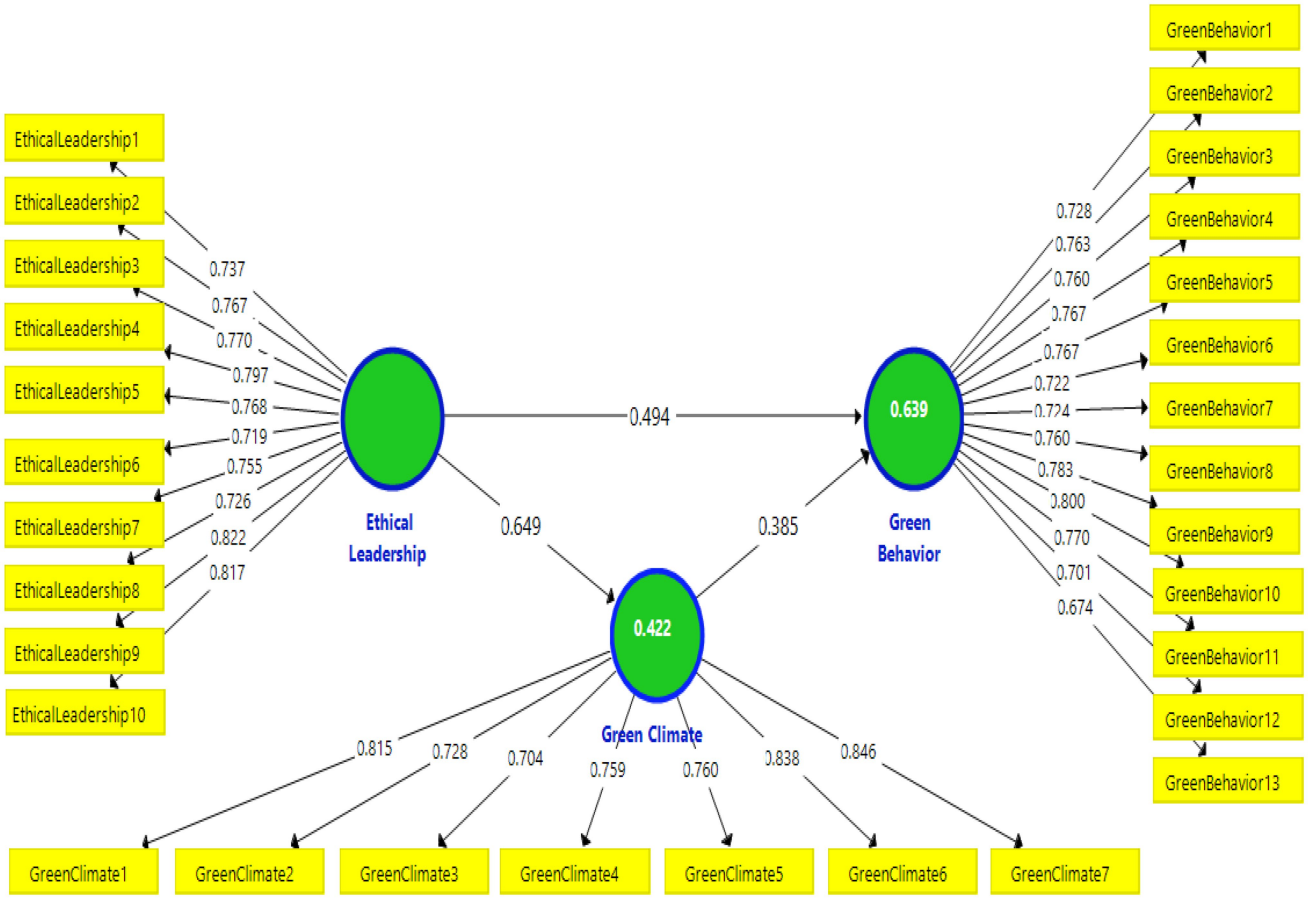
Adjusted Measurement Mode.

A threshold value of ≥0.7 for each item’s loading is considered reliable (Hair et al., 2017), but loading greater than 0.6 is adequate when outer loadings have high scores of loadings to match Average Variance Extracted (AVE) and Composite Reliability (CR; Ramayah et al., 2018). Thus, only one item (Green Climate8 = in our hotel, employees care about the environment) is found short of the required minimum level and is removed. In addition, the adjusted measurement model of this study is highlighted in Figure 4.

Table 3 highlights the construct’s validity and reliability, thereby showing that the constructs have high validity and reliability (Hair et al., 2010b).

**Table 3:** Validity and reliability.

**Construct**	**Cronbach’s alpha**	**rho_A**	**CR**	**AVE**
ESEL	0.923	0.924	0.935	0.591
EGB	0.934	0.935	0.943	0.561
PGC	0.892	0.897	0.916	0.609

In Table 4, the bold values (square root of AVE) are more than the correlation value between the constructs, indicating an adequate discriminant validity (Fornell and Larcker, 1981).

**Table 4:** Fornell Larcker criterion.

Construct	ESEL	EGB	PGC
ESEL	0.769		
EGB	0.744	0.749	
PGC	0.649	0.706	0.78

In Table 5, the item loadings are higher than the cross-loadings, thus establishing discriminant validity (Chin, 1998).

**Table 5:** Cross loading.

Item	ESEL	EGB	PGC
Ethical Leadership1	0.737	0.495	0.399
Ethical Leadership2	0.767	0.514	0.446
Ethical Leadership3	0.77	0.584	0.45
Ethical Leadership4	0.797	0.576	0.497
Ethical Leadership5	0.768	0.61	0.493
Ethical Leadership6	0.719	0.581	0.5
Ethical Leadership7	0.755	0.585	0.494
Ethical Leadership8	0.726	0.57	0.606
Ethical Leadership9	0.822	0.598	0.515
Ethical Leadership10	0.817	0.582	0.553
Green Behavior1	0.579	0.728	0.534
Green Behavior2	0.625	0.763	0.602
Green Behavior3	0.531	0.76	0.563
Green Behavior4	0.616	0.767	0.53
Green Behavior5	0.61	0.767	0.559
Green Behavior6	0.528	0.722	0.531
Green Behavior7	0.518	0.724	0.483
Green Behavior8	0.522	0.76	0.538
Green Behavior9	0.541	0.783	0.552
Green Behavior10	0.552	0.8	0.517
Green Behavior11	0.494	0.77	0.55
Green Behavior12	0.584	0.701	0.436
Green Behavior13	0.515	0.674	0.449
Green Climate1	0.498	0.487	0.815
Green Climate2	0.353	0.425	0.728
Green Climate3	0.62	0.675	0.704
Green Climate4	0.483	0.531	0.759
Green Climate5	0.508	0.51	0.76
Green Climate6	0.523	0.584	0.838
Green Climate7	0.485	0.563	0.846

In Table 6, the values are less than 0.9, thus establishing discriminant validity (Henseler et al., 2015). Table 7 shows that the values of VIF are less than 5, hence highlighting the non-existence of multicollinearity (Field, 2009).

**Table 6:** HTMT.

Construct	ESEL	EGB	PGC
ESEL			
EGB	0.796		
PGC	0.696	0.756	

**Table 7:** Collinearity assessment.

Dependent Construct	Predictor construct	Collinearity (VIF < 5)
EGB	ESEL	1.730
	PGC	1.730
PGC	ESEL	1

### Structural Model

The values of the structural model (direct relationships) of this research are highlighted in Table 8, indicates that ethical leadership is positively and significantly related to green behavior (*β* = 0.494, *t* = 7.469, *p* < 0.05), thus, supporting the first hypothesis. Furthermore, ethical leadership is positively and significantly related to green climate (*β* = 0.649, *t* = 18.419, *p* < 0.05), thus, supporting the second hypothesis. In addition, the green climate has a positive and significant effect on green behavior (*β* = 0.385, *t* = 6.257, *p* < 0.05), thus supporting the third hypothesis (see Table 8).

**Table 8:** Structural model direct hypotheses results.

Hypotheses	Relationship between the constructs	Original sample (O)	Sample mean (M)	T statistics (|O/STDEV|)	Values of *p*	Decision
H1	ESEL➔EGB	0.494	0.494	7.469	0.000	Supported
H2	ESEL➔PGC	0.649	0.652	18.419	0.000	Supported
H3	PGC➔EGB	0.385	0.386	6.257	0.000	Supported

The following Table 9 shows the results of the coefficient of the determinant (*R*^2^), which are at a moderate level; for instance, *R*^2^ values of 0.75, 0.50, and 0.25 are known to be substantial, moderate, and weak, respectively (Hair et al., 2017).

**Table 9:** Coefficient of the determinant (*R*^2^).

Construct	R Square	R Square adjusted	Explanatory power
EGB	0.639	0.636	Moderate
PGC	0.422	0.419	Moderate

Scholars identified that the *f* ^2^ values of 0.02, 0.15, and 0.35 are considered a small, medium, and large effect sizes, respectively (Cohen, 1988). Table 10 indicates the results of the *f* ^2^.

**Table 10:** *f*^2^—effect size to *R*^2^.

Construct	EGB		PGC	
	*f* ^2^	effect	*f* ^2^	effect
ESEL	0.390	Large	0.730	Large
PGC	0.237	Medium		

Hair et al. (2017) have identified that the recommended values of *Q*^2^ are 0.35 (large), 0.15 (medium), and 0.02 (small), indicating the level of the predictive relevance for endogenous construct. The following Table 11 shows the results of *Q*^2^.

**Table 11:** Predictive relevance (*Q*^2^).

Endogenous latent construct	*Q* ^2^	Level of predictive relevance
EGB	0.348	Medium
PGC	0.241	Medium

After conducting the bootstrapping analysis, results show that PGC plays a mediating role between ESEL and EGB in the hotel industry of Pakistan (see Table 12).

**Table 12:** Result of mediation hypotheses (indirect effects).

Hypotheses	Indirect effect	Original sample (O)	Sample mean (M)	T statistics (|O/STDEV|)	Value of *p*	2.50%	97.50%	Decision
H4	ESEL➔PGC➔EGB	0.250	0.251	6.047	0.000	0.169	0.336	Supported

**Figure 5:**
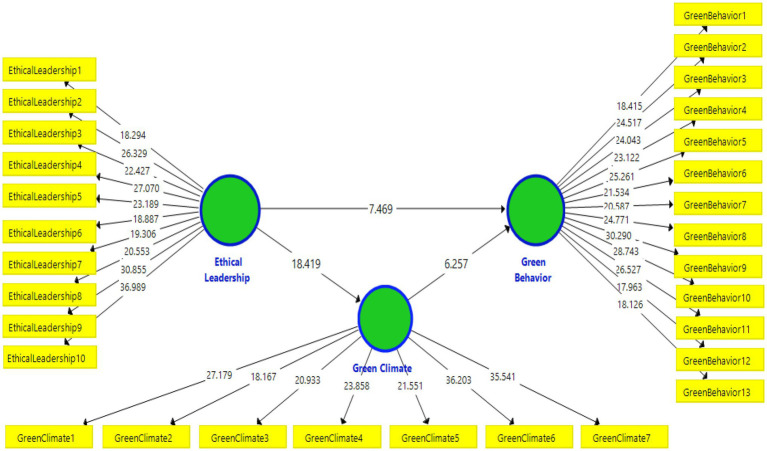
PLS Bootstrapping results for the structural model.

The following Figure 5 highlights the results of the bootstrapping analysis.

## Findings, Discussion, and Theoretical Implications

Employee green behavior is being researched (Dumont et al., 2017; Saeed et al., 2019; Suganthi, 2019), and its antecedents are being investigated. Thus, the relationship between environmental-specific ethical leadership conduct, psychological green climate, and staff green behavior is examined in this study. ESEL was positively connected to PGC, ESEL was positively related to EGB, PGC was positively associated with EGB, and PGC mediates between ESEL and EGB, supporting the current research hypothesis. As employees observe and learn desirable and expected behaviors from them. As a result, when ESEL is in place in a company, subordinates learn the proper way to behave through role modeling, which involves observing their boss’s attitude and behavior (Yasir and Rasli, 2018). As a result, when subordinates think that their leaders are concerned about sustainable development, a green psychological climate is created, which encourages employees to engage in green behavior. As a result, one of the underlying processes by which ethical leadership is linked to green behavior in Pakistan’s hotel business is a green psychological environment.

Previous literature shows that ethical leadership has a positive relationship with employees’ pro-social and ethical behaviors and a negative relationship with unethical behaviors (Brown et al., 2005; Lu and Lin, 2014; Aryati et al., 2018; Zhang and Tu, 2018; Liu and Zhao, 2019; Yasir and Khan, 2020), suggesting that ethical leadership can promote ethical and pro-environmental behaviors. However, limited literature exists on the relationship between ethical leadership and green behavior (Saleem et al., 2020). Thus, this research extends the existing knowledge related to ethical leadership and green behavior as it shows a positive association between the two constructs. This study also extends the current literature on psychological green climate. This study highlights that green climate plays a significant mediating role between ethical leadership and green behavior precisely in Pakistan’s hotel industry. Thus, this study highlights that ethical leaders consider protecting the natural environment a moral obligation (Wu et al., 2015). Because ethical leaders set ethical standards in an organization (Creswell and Creswell, 2017), thereby shaping subordinates shared perception that the organization’s policies and procedures are pro-environmental, this perception leads to inspiring subordinates to engage in green behavior (Saleem et al., 2020). Hence, an ethical leader plays a vital role in establishing and fostering a green psychological climate, motivating employees to engage in green workplace behavior.

### Practical and Managerial Implications

This research has several practical implications for hoteliers that want to encourage green behavior among their employees. Because subordinates prefer to follow their leaders’ behavior, the current study implies that managers as role models establish examples of pro-environmental behavior. Furthermore, these managers should develop, promote, and implement green policies, thereby influencing subordinates’ perceptions of the organization’s policies, procedures, and practices as environmentally friendly. Hoteliers, for example, should prioritize environmental protection, which includes actions such as emission reduction and waste minimization, recycling, water and energy conservation, and eco-friendly purchasing strategies (Lee et al., 2010; Stalcup et al., 2014). Furthermore, hoteliers must motivate and persuade workers to execute green projects by implementing an incentive scheme, such as offering money and non-financial incentives (Al-Romeedy, 2019). Such measures will create a psychological green atmosphere, which is a crucial determinant of employees’ green behavior. Furthermore, hoteliers must attract and retain individuals who care about the environment and provide training and development programs for employees, thereby communicating the importance of environmentally specific ethical leadership behavior, which can help foster a psychological green climate conducive to employee green behavior. As a result, the current study demonstrates the importance of environmental-specific ethical leadership conduct and a green climate as antecedents of employee green behavior in Pakistan’s hotel business.

### Research Limitations and Future Directions

First, a sample of the current research was from the hotel industry in Pakistan. Thus, caution about the generalization of the present study’s findings might be taken. In addition, future research should consider dissimilar cultures and industries for the understudy framework, for instance, the manufacturing industry, banking, and telecommunication sector. Second, the current study is cross-sectional, and this study design does not allow for a determination of the direction of causality between the constructs. Therefore, a longitudinal study is needed, thereby establishing the causal directions of the understudy framework. Third, the current study investigated psychological green climate as a mediator. However, other underlying psychological mechanisms may also explain the link between ESEL and EGB. Therefore, future research may explore other mediating mechanisms that might clarify the underlying psychological mechanisms through which ESEL is related to EGB. Lastly, this research had an acceptable sample size. However, it seems to be relatively small. Therefore, future studies should utilize a somewhat larger sample size.

## Conclusion

Organizations are increasingly adopting green human resource management policies and practices to encourage environmentally friendly behaviors. Research shows that adopting green policies and practices is also beneficial for the hospitality sector. Therefore, this study investigated the mediating role of psychological green climate between environmental-specific ethical leadership and employees’ green behavior, specifically in the context of the hotel industry of Pakistan. The findings confirmed that PGB mediates the relationship between ESEL and EGB in the hospitality industry of Pakistan. This study provides several practical implications of value to hoteliers willing to promote employee green behavior. Team leaders and managers must understand their role as role models and set examples of pro-environmental behavior because subordinates tend to imitate their leader’s behavior. Furthermore, these managers should establish, promote, and implement pro-environmental policies and initiatives. For instance, hoteliers should focus on protecting the natural environment, thereby involving such activities as reducing emission and waste minimization, recycling, saving water and energy, and implementing eco-friendly purchasing policies.

## Data Availability Statement

The original contributions presented in the study are included in the article/Supplementary Material; further inquiries can be directed to the corresponding author.

## Author Contributions

NUK: conceptualization, formal analysis, investigation, writing—original draft preparation, and project administration. JC and MY: methodology and software. JC: resources and funding acquisition. RAS, NCN, MY, and HAB: writing—review and editing. NUK: project administration. All authors have read and agreed to the published version of the manuscript.

## Funding

The key project of humanities and social sciences of Anhui Provincial Department of Education. Funding number: SK2020A0595.

## Conflict of Interest

The authors declare that the research was conducted in the absence of any commercial or financial relationships that could be construed as a potential conflict of interest.

## Publisher’s Note

All claims expressed in this article are solely those of the authors and do not necessarily represent those of their affiliated organizations, or those of the publisher, the editors and the reviewers. Any product that may be evaluated in this article, or claim that may be made by its manufacturer, is not guaranteed or endorsed by the publisher.
